# The association of park use and park perception with quality of life using structural equation modeling

**DOI:** 10.3389/fpubh.2023.1038288

**Published:** 2023-01-25

**Authors:** Hanish P. Kodali, Emily B. Ferris, Katarzyna Wyka, Kelly R. Evenson, Joan M. Dorn, Lorna E. Thorpe, Terry T.-K. Huang

**Affiliations:** ^1^Center for Systems and Community Design, Graduate School of Public Health & Health Policy, City University of New York, New York, NY, United States; ^2^Department of Epidemiology, Gillings School of Global Public Health, University of North Carolina-Chapel Hill, Chapel Hill, NC, United States; ^3^Department of Community Health and Social Medicine, City University of New York School of Medicine, New York, NY, United States; ^4^Department of Population Health, School of Medicine, New York University, New York, NY, United States

**Keywords:** quality of life, structural equation model, perception of neighborhood park, built environment, New York City, low-income neighborhoods, perceived stress, park use

## Abstract

**Introduction:**

The literature is limited on the impact of neighborhood parks on quality of life (QoL) and the mechanism linking them.

**Methods:**

In this paper, we applied the structural equation model to data from a cross-sectional sample of 650 participants in low-income communities of New York City, we examined the associations of neighborhood park use vs. park perception and QoL, and whether these associations were mediated through self-reported perceived stress. We also examined whether park use mediated the relationship between park perception and QoL.

**Results:**

We found that park use had a significant but weak association with QoL (standardized *β* = 0.08, 95% confidence interval (CI): 0.02, 0.15, *p* = 0.02), but this relationship was not mediated by self-reported stress. Park perception was more strongly associated with QoL than park use (standardized *β* = 0.23, 95% CI: 0.16, 0.30, *p* < 0.01), and this was partly mediated by self-reported stress (indirect effect- standardized *β* = 0.08, 95% CI: 0.03, 0.13, *p* < 0.01) and, to a lesser extent, by park use (indirect effect- standardized *β* = 0.01, 95% CI: 0.00, 0.02, *p* = 0.01).

**Discussion:**

Having well-perceived parks appears to be an important factor for QoL independent of park use, suggesting that quality parks may benefit everyone in a community beyond park users. This strengthens the argument in favor of increasing park investment as a strategy to improve population wellbeing.

## 1. Introduction

Quality of life (QoL) is increasingly put forward as a key health outcome measure in its own right, as the notion of wellbeing is increasingly recognized as more than economic wealth or the absence of clinical disease (1). The European Commission has in recent years called for the inclusion of measures of QoL in the context of sustainable development (2). Other international organizations, such as the Organization for Economic Co-operation and Development has similarly produced the Better Life Index, consisting of measures of material living conditions as well as QoL (3). In public health research, there has been a rise in the use of QoL measures to evaluate policies (4–6) or compare the wellbeing of populations in different countries (7–9). However, beyond ecological comparisons, much of the research on QoL only explores associations with specific health conditions. Little research has focused on environmental correlates and determinants that might contribute to QoL.

Parks and green space are thought to play an important and positive role in health outcomes, including in QoL, especially in urban environments (10–14). While prior research on parks or green space has focused more on health behaviors such as physical activity or conditions such as obesity or depression (14), emerging research shows that frequency of park use may also be positively associated with QoL in China (13) and Turkey (11). Among United States (US) college students, frequent and active use of green space has also been positively associated with QoL (15). Another study conducted in Denmark showed that people who lived closer to green space reported better health-related QoL (16). However, the existing evidence base on QoL as an outcome remains limited and the mechanisms by which parks may exert a positive effect on QoL is still unclear.

In addition to studies that focus on the frequency of park use or proximity to parks in relation to QoL, an emerging body of research shows that subjective perceptions of parks may be an important consideration. For instance, higher QoL has been associated with more positive perceptions of neighborhood open spaces (17), area green neighborhood qualities (18), and greenway trails (19). Interestingly, a study in Hungary compared objective spatial indicators and visitors' perceptions of urban parks, and found that the two types of measures were only moderately correlated, highlighting the potential importance of both types of measures in research (20). Another study in Australia also showed that perceived greenness of the community environment did not necessarily correlate well with objectively measured greenness (21). Indeed, perceptions of parks may not directly translate into park usage (22), and the relative role of park perception vs. park use in QoL has not been well-studied.

One possible pathway linking park exposure to QoL may be perceived stress. Several studies have demonstrated that parks could play a role in reducing stress (23, 24). For example, Tyrväinen et al. (25) showed that exposure to nature areas was associated with lower stress levels compared to exposure to built-up areas. Similarly, another showed that exposure to nature vs. urban street conditions resulted in greater restorative recovery from stress even after adjusting for stress reactivity (26). However, findings on the role of stress have not always been consistent (27), and the extent to which it mediates the association of park exposure or perception with QoL is yet unclear.

In light of the fact that QoL has not been well-studied in relation to park exposure variables, and that there is lack of clarity on the relative role of park use vs. park perception and the mechanism connecting these to health outcomes, this paper sought to address these gaps through a cross-sectional structural equation modeling (SEM) study. We also tested the mediational effect of perceived stress and whether park use mediated the association between park perception and QoL. We used data from predominately low-income minority communities in New York City (NYC), a population that has been understudied in the built environment literature.

## 2. Materials and methods

### 2.1. Data

We used baseline data from the Physical Activity and Redesigned Community Spaces (PARCS) Study, conducted across 54 community parks throughout NYC during 2016-18 (28). PARCS is an ongoing study examining the relationship between parks and wellbeing. Study parks were located in neighborhoods that met at least two of three criteria: high poverty (≥20% population below the poverty line), high population growth (25% growth 2000–10), and high population density (≥110 people/acre).

Individual-level data in the PARCS Study were obtained from residents that lived within a 0.3-mile Euclidean buffer around each neighborhood park. All participants were recruited by convenience sampling. They were over 18 years old, lived in the study neighborhoods for at least 2 years, intended to stay in the neighborhood for at least 4 years, spoke English, Spanish or Chinese, and had no mobility limitations. At baseline, participants responded to a survey on park perception and usage and psychosocial and community wellbeing. Only participants who provided consent were enrolled in the study. The study was approved by the City University of New York Institutional Review Board.

### 2.2. Measures

#### 2.2.1. Outcome variable

##### 2.2.1.1. Quality of life

QoL was operationalized using a 9-item scale with Likert response options from the public health surveillance wellbeing scale (29). It was developed and validated by the Centers for Disease Control and Prevention (CDC), with an original Cronbach's alpha of 0.87 and good construct validity. A 10th item from the original CDC scale, that asked respondents to indicate the number of days they felt very healthy and full of energy, was dropped because it was not based on Likert-type responses and the concepts of feeling healthy and energetic were already captured in other items.

The 9 items of the scale captured 3 major domains of wellbeing: mental (5 items), social (2 items), and physical health (2 items). The items related to satisfaction with life, clear sense of purpose, and feeling accomplished were rated on a 5-point Likert scale, ranging from 1 = “strongly disagree” to 5 = “strongly agree.” Feelings of cheerfulness and hopelessness in the last 30 days were rated between 1 = “none of the time” to 5 = “all the time.” Items on satisfaction with one's energy level, family, friends, and social life were rated on a scale of 1 (very dissatisfied) to 10 (very satisfied). Lastly, self-reported overall health was rated between 1 (excellent) to 5 (poor). The feeling of hopelessness and self-reported health status were reverse coded so that higher values meant higher QoL for all items.

#### 2.2.2. Exposure variables

##### 2.2.2.1. Frequency of park use

To assess the frequency of park use, we adopted 2 questions from Veitch et al. [test-retest intraclass correlation (ICC) = 0.79–0.85] (30). The original questions pertained to park use in the past 3 months; however, we modified this to the past 30 days to reduce recall bias. The two questions were: “In the past 30 days, on average, how often have you visited the study park (there was only one study park in each neighborhood)?” and “In the past 30 days, on average, how often have you visited a park other than the study park?” Response options for each question included “daily,” “4–6 times/week,” “2–3 times/week,” “once/week,” “2–3 times/month,” “once/month,” “less than once/month,” and “have not visited in past 30 days.” Based on a previously developed methodology (31), a random number was computer-generated within the range of each category as the number of days a person visited a park. The highest frequency of park use between the 2 variables was used as a proxy for park use by a given participant in his or her neighborhood.

##### 2.2.2.2. Park perception

For individual-level park perception, we used a set of 10 survey questions developed by Veitch et al. (test-retest ICC = 0.36–0.72) (30). Items were rated on a 5-point Likert scale between 1 = “strongly disagree” and 5= “strongly agree.” These items asked about the attractiveness, safety, maintenance, shade availability, and dog-walking facilities in neighborhood parks. In addition, participants rated the overall quality of parks in the neighborhood and whether children liked going to these parks.

#### 2.2.3. Mediator

##### 2.2.3.1. Self-reported stress

To assess stress, we used the Perceived Stress Scale (10 items) by Cohen et al. (Cronbach's alpha = 0.78) (32). The scale measured stress experienced by the participants in the past 30 days on a 5-point Likert scale (0 = “never” to 4 = “very often”). The total score ranges from 0 to 40, with higher values indicating high perceived stress.

#### 2.2.4. Covariates

Participants self-reported age (years), sex (female vs. male), household income (below and above $20,000 per annum), and race/ethnicity (Latino, Black, White, and Other).

### 2.3. Statistical analysis

We conducted a multiple-mediation analysis using an SEM with complete cases. SEM provides an advantage since the models include specific and accumulative associations between different variables and evaluate multiple mediators effects simultaneously in a single model (33). Additionally, SEM tackles concerns related to measurement errors in the survey datasets better than regression models (34). We developed latent constructs for two measures, park perception, and QoL. Both measurement models were further assessed by confirmatory factor analysis. Factor loadings were used to check the association between the latent constructs and their observed variables. Observed variables with factor loadings <0.40 were excluded.

In addition to the direct effect of park use and park perception on QoL, we evaluated 3 mediation pathways: (35) (1) the effect of park use on QoL, as mediated by stress (park use → stress → QoL), (2) the effect of park perception on QoL, as mediated by stress (park perception → stress → QoL), and (3) the effect of park perception on QoL, as mediated by park use (park perception → park use → QoL). The bootstrap method, based on 1,000 re-samples, was used to generate standard errors and significance tests for individual parameters. All the ordinal variables were recoded so that the lowest value started from zero. Standardized total, direct and indirect path coefficients (β), along with the 95% bias-corrected percentile confidence interval (CI) and *p*-value were reported. To validate the fitness of the SEM, χ^2^ values, the Comparative Fit Index (CFI), the Tucker-Lewis Index (TLI), and the root-mean-square error of approximation (RMSEA) were used. We considered a model acceptable when the CFI and TLI were 0.90 or greater and the RMSEA value was 0.06 or less.

Additionally, for sensitivity analysis, we used the Full Information Maximum Likelihood (FIML) approach to impute the missing values for those participants who had answered at least one but not all of the survey questions and re-ran the SEM with the larger sample (*n* = 1,354). We chose FIML over the other imputation methods since it provides better estimates for SEM (36, 37).

R statistical software v.3.6.2 (38) and IBM SPSS AMOS v.26.0 (39) were used for analysis. Alpha was set at 0.05.

## 3. Results

### 3.1. Sample characteristics

Participants in the sample (*n* = 650) had a mean age of 38.8 ± 11.8 years, half (53.4%) had an annual household income below $20,000, and 81.7% were women. Latinos constituted 48.8% of the sample, while 38% were Black (Table 1). On an average, the participants in our study sample visited their neighborhood parks ~12.1 ± 10.3 days in the past 30 days and they reported moderate stress (24.3 ± 7.5).

**Table 1:** Socio-demographic and health characteristics of the study participants.

	***n*** **(%)**
**Gender**	
Male	119 (18.3)
Female	531 (81.7)
**Race/ethnicity**	
Latino	317 (48.8)
Non-Latino Black	247 (38.0)
Non-Latino White	33 (5.1)
Non-Latino Other	53 (8.1)
**Household income per year**	
≥$20,000	303 (46.6)
< $20,000	347 (53.4)
	**Mean (SD)**
**Age (years)**	38.8 (11.8)
**Park use in last 30 days (# of days)**	12.1 (10.3)
**Perceived stress scale score**	24.3 (7.5)

### 3.2. Confirmatory factor analysis of latent constructs

Means and standard deviations of underlying factors of the latent constructs of QoL and park perception are shown in Table 2. Table 2 also shows the confirmatory factor analysis results of the park perception and QoL scales, including all variables with factor loadings ≥0.40. This resulted in 0 of 9 variables excluded from QoL and 3 of 10 variables being excluded from park perception. Cronbach's alpha was 0.84 for QoL and 0.86 for park perception.

**Table 2:** Confirmatory factor analysis of the latent constructs of park perception and quality of life.

**Latent variable**	**Measured variable**	**Mean (SD)**	**Factor loading**
Park perception	•I am satisfied with overall quality of parks in my neighborhood	1.95 (1.17)	0.78
(Cronbach's Alpha = 0.86)	• Parks in my neighborhood are attractive	1.88 (1.19)	0.77
	• Parks in my neighborhood are safe	1.92 (1.14)	0.78
	• Parks in my neighborhood are well maintained	1.94 (1.17)	0.76
	• Parks in my neighborhood have satisfactory shade	1.97 (1.16)	0.6
	• Parks in my neighborhood have suitable dog walking facilities	1.38 (1.21)	0.56
	• My children like going to parks in my neighborhood	2.87 (1.10)	0.44
Quality of life	Mental health		
(Cronbach's Alpha = 0.84)	• I am satisfied with my life…	2.71 (1.11)	0.61
	• My life has a clear sense of purpose…	3.08 (1.13)	0.64
	• Most days I feel a sense of accomplishment from what I do…	2.92 (1.12)	0.66
	• How much of the time during the past 30 days have you felt…? Cheerful…	2.61 (0.96)	0.68
	• How much of the time during the past 30 days have you felt…? Hopeless…	2.76 (1.13)	0.51
	Social health		
	• How satisfied you are with each of the following items, Your family life…	6.91 (2.25)	0.59
	• How satisfied you are with each of the following items, Your friends and social life…	5.64 (2.56)	0.58
	Physical health		
	• How satisfied you are with each of the following items, Your energy level…	5.68 (2.54)	0.66
	• In general, would you say your health is:	2.28 (1.04)	0.41

### 3.3. Structural equation model

**Figure 1:**
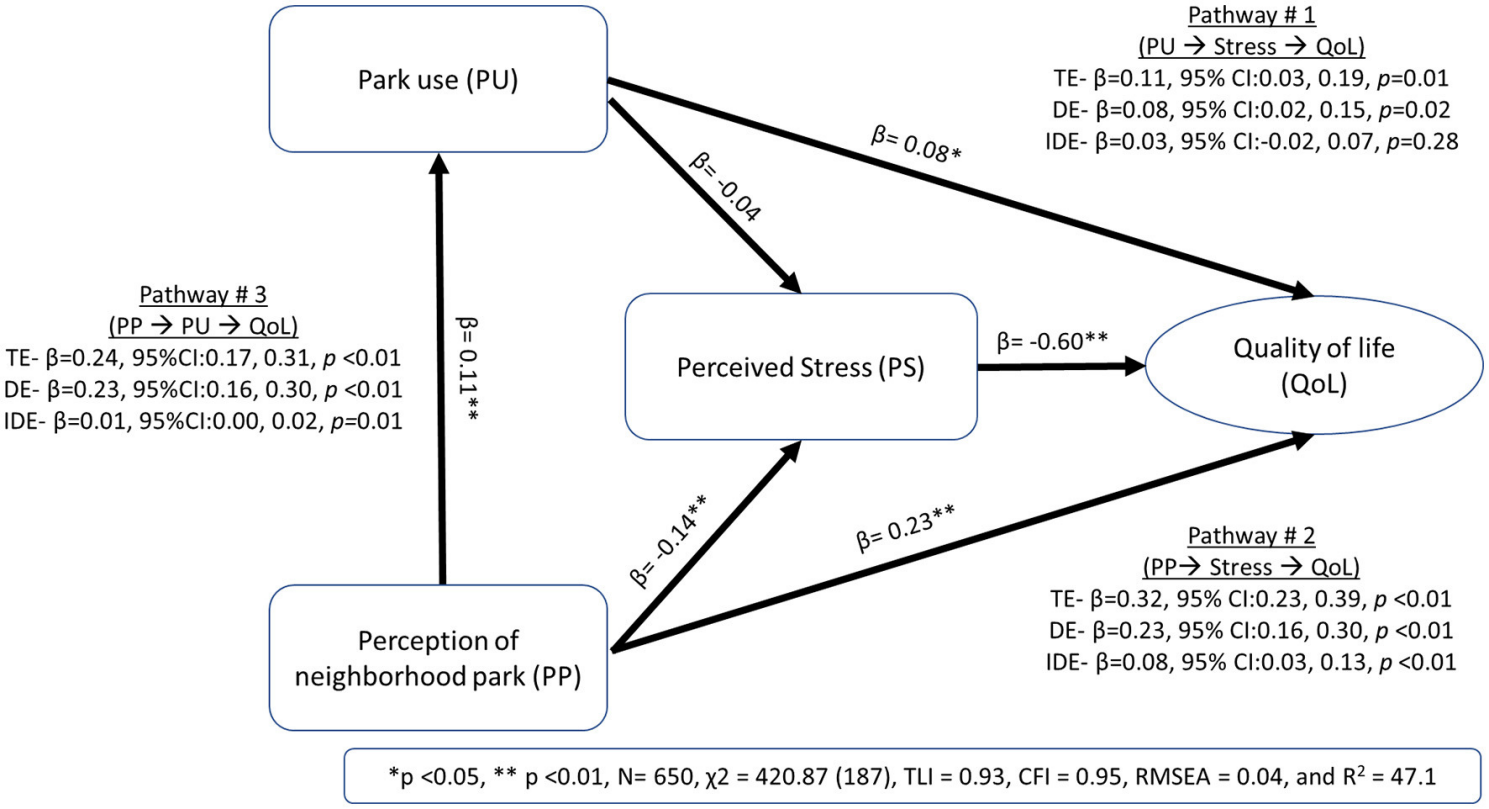
Structural equation model of park use, park perception, and self-reported stress in relation to quality of life. *β*, Standardized coefficient; *p, p*-value; CI, Confidence interval; TE, Total effect; DE, Direct effect; IDE, Indirect effect; *χ*^2^, Chi-square value; CFI, Comparative Fit Index; TLI, Tucker-Lewis Index; RMSEA, root-mean-square error of approximation.

**Figure 2:**
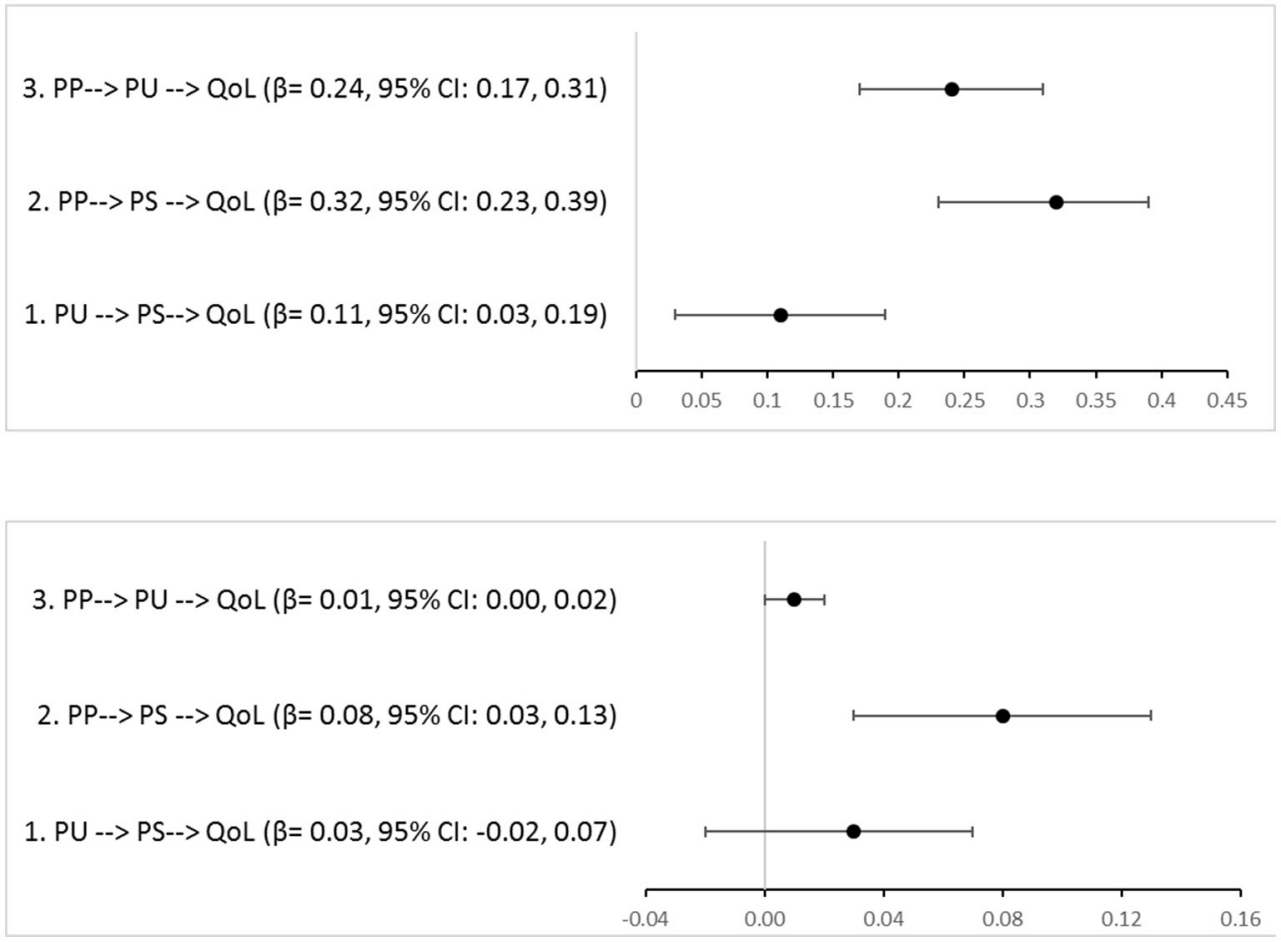
Forest plot of the total **(top)** and indirect **(bottom)** effect of park use, park perception on quality of life via self-reported stress. *β*, standardized coefficient; CI, confidence interval; PU, park use; PP, perception of park; PS, perceived stress; QoL, quality of life.

The final SEM showed a good model fit with χ^2^ = 420.87 (187), TLI = 0.93, CFI = 0.95, and RMSEA = 0.04. The model explained 47.1% of the variance in QoL. Figure 1 shows the total, direct and indirect effects in the three mediation analyses. For Pathway #1, park use showed a significant direct association with QoL (standardized β = 0.08, 95% CI: 0.02, 0.15, *p* = 0.02), with no significant mediation through self-reported stress (standardized β = 0.03, 95% CI: −0.02, 0.07, *p* = 0.28). In Pathway #2, park perception had a direct association with QoL (standardized β = 0.23, 95%CI: 0.16, 0.30, *p* < 0.01) and a significant indirect association that was partially mediated through self-reported stress (standardized β = 0.08, 95% CI: 0.03, 0.13, *p* < 0.01). In Pathway #3, park perception also had a significant indirect association with QoL that was mediated through park use (standardized β = 0.01, 95% CI: 0.00, 0.02, *p* = 0.01). Similar results were found when SEM was performed with multiple imputations for missing values in the larger sample (*n* = 1,354; data not shown). Figure 2 shows the forest plots of the total and indirect effects.

## 4. Discussion

To our knowledge, this is the first study to examine the role of park use and park perception on QoL and to explore the mechanisms that may underlie these relationships. Park perception had a stronger association with QoL than park use. In addition, the association of park perception with QoL was mediated through self-reported stress to a greater extent than through park use. Although the model tested was relatively parsimonious, it explained nearly half of the variance in QoL.

The significant association between park use and QoL in our study was corroborated by prior research in urban settings showing that visits to parks or green space improved mental and physical health (40). Of note, our QoL measure also encompassed social domain of health, suggesting that the benefit of parks may be generalized to a broader conceptualization of wellbeing. That said, it was unexpected that the association of park use with QoL was not mediated by stress, despite the fact that stress was strongly associated with QoL. This may suggest that park use may not directly translate into a reduction in perceived stress and its effect on QoL may be due to other factors. For example, park use may increase social interaction or physical activity (10), which can have an impact on immune and endocrine functions, including the release of endorphins (41–43). Endorphins, in turn, may mitigate depressive symptoms (44, 45). In addition, there is some evidence, at least in animal studies, that chronic stress may dampen the effect of reward in the brain, suggesting that stress may moderate rather than mediate the relationship between park use and QoL (46, 47). More research on such alternative pathways is warranted. It is also possible that the effect of park use on stress can only be observed following long term use of park, which our study was not able to examine.

That perception of parks may play a larger role in QoL than actual park use, as indicated from the SEM. This suggests that having quality parks in a neighborhood may confer benefits to the wellbeing of local residents even if they do not engage in frequent park use. Although this is a new finding, the National Recreation and Park Association previously conducted a survey on the perception and use of local recreation and park services among Americans and showed that even non-users of parks believed that having a local park, playground, or open space in their neighborhood was important to their personal and community wellbeing (48). In another study of Switzerland adults, higher satisfaction with the living environment and perception of access to green spaces were associated with higher health-related QoL (49).

To our knowledge, there have been no studies that specifically examined the role of park perception on QoL *via* the mediation of stress. However, in a virtual reality experiment, it was reported that parks and urban green space reduced stress levels through olfactory, audio, and visual sensory stimulation (50). Studies indicate that exposure to (without necessarily requiring interaction with) urban green space could help reduce stress and improve mental wellbeing or QoL (51–55). Of note, in our study, there was a potential effect of park perception on QoL beyond the mediating role of stress, suggesting that there are likely other mechanisms that need further research.

While we found an association of park perception with QoL, which was partially mediated by park use, it was notable that this mediation was weak. On one hand, some research has shown that park perception is correlated with park use (56). On the other hand, a study of young people in three cities in China found that while a perception of green space accommodating health promotion activities was associated with an increased willingness to use the park, it had less of an effect on actual park usage (22).

QoL is an increasingly important and recognized population outcome globally. For decades, countries have used economic indicators such as gross domestic product (GDP) to measure progress and prosperity. However, GDP does not capture environmental and social wellbeing or the degree to which people are satisfied with their living conditions (2, 57–61). In 2020, New Zealand released a “wellbeing budget,” with spending and policy decisions based on citizens' health and wellbeing rather than GDP or economic growth (59). Our study uses a robust yet simple measure of QoL that was previously validated by the CDC. Our findings contribute to the growing body of literature on the importance of QoL and what cities and countries can do to improve such an outcome.

In general, urban green space shares a complex relationship with health. It is inversely associated with the prevalence of non-communicable diseases related morbidity, and mortality (14, 62–64), premature death (65, 66), and stress (23, 24). It is also positively associated with improved pregnancy outcomes (67), mental health (68, 69), and life expectancy (70). In a broader sense, green space may influence health through five major pathways: physical, mental, social, environmental, and economic. It provides space for physical activity, which reduces the burden of various non-communicable diseases (14, 62, 63). Exposure to green space reduces stress, anxiety, depression, and various mental health issues (71). It also promotes social interaction and cohesion and improves social capital (72). Green space also reduces heat and air pollution, impacting environmental health (73). Last but not least, the presence of green space improves real estate values (74) and provides opportunities for jobs and small businesses (72). All these outcomes interact to contribute to individual and community QoL, but research is warranted to examine these different pathways in a synergistic and systems framework.

This study could be beneficial to urban planners and policymakers looking to revitalize or future-proof urban environments. Currently, there is a rise in investment in urban green space to create more equitable parks, with special attention paid to building new parks or renovating existing parks in low-income neighborhoods (75). It is essential to understand a community's perception of parks during redesign and renovation. For instance, in recent years, the City of Chicago has also invested more than US$44 million to repair over 300 playgrounds. Interestingly, the Chicago experience showed that the renovation led to increased park use in higher-income neighborhoods, suggesting the impact might not have been equitable and those future efforts would need to do better in aligning park redesign with community preferences and participation (76). These lessons were shared in New York City, where since 2014, the NYC Department of Parks and Recreation has been implementing the Community Parks Initiative, investing over US$300 million to redesign and renovate 67 small parks in underserved neighborhoods (77). As part of the process, the city organized community input meetings to understand the needs of diverse potential park users. Building on this effort, our team is now deepening the engagement with select communities to co-design park-based strategies to further enhance the social environment of neighborhoods that have recently experienced park renovation.

The primary limitation of our study is that it was cross-sectional. Therefore, caution should be taken in inferring causality. Future longitudinal studies could be helpful to examine cause-and-effect and to determine if our findings hold over time. Second, our study did not examine the multiple pathways that parks could influence QoL. However, this paper sets the stage for future research that can account for the diverse synergistic framework described by the World Health Organization (72). In addition, our study was conducted in NYC, which can be quite different than other cities in the US and globally. Thus, the generalization of our findings to other locales may be limited. Lastly, we would like to acknowledge that our study population was mostly female (~82%). Future research will need to examine whether there are gender differences in the relationship between parks and QoL.

Nevertheless, our study also has many strengths. First, in light of the issue of environmental and health equity, we specifically undertook our study in low-income, minoritized communities, making an important contribution to the limited literature on the topics of parks and QoL in underserved populations. In addition, the use of SEM in this area of inquiry was novel and allowed us to test the effect of multiple pathways involving latent constructs simultaneously (33, 35). Last but not least, our paper makes an important contribution by establishing the important role of park perception in health beyond park access or use.

## 5. Conclusions

In conclusion, we showed that both the perception and frequency of use of parks are important to QoL. However, our findings also suggest that having quality parks in a neighborhood could be broadly beneficial to community residents, even beyond frequency of use. This finding strongly supports the investment in community parks, as all residents may benefit from such investment, not just regular park users. In addition, our study showed that stress reduction may be an important mechanism for the effect of park perception on QoL, but that it may not play a role in associations between park use and QoL. Nonetheless, our model suggests that this explanation may be incomplete, and more research is needed to further elucidate the ways in which park perception and park use contribute to QoL. With parks and QoL both at the forefront of policy discussions in the US and around the world, this study demonstrates that these two factors are indeed connected and suggests that strategic investment in public parks can play a critical role in reducing inequities and improving the wellbeing of all residents.

## Data availability statement

The raw data supporting the conclusions of this article will be made available by the authors, without undue reservation.

## Ethics statement

The studies involving human participants were reviewed and approved by City University of New York Institutional Review Board (#2016-0248). The patients/participants provided their written informed consent to participate in this study.

## Author contributions

HK: methodology, software, formal analysis, data curation, writing—original draft, and visualization. EF: writing—original draft. KW: supervision. KE and JD: writing—review and editing. LT: writing—review and editing and funding acquisition. TH: conceptualization, methodology, writing—review and editing, supervision, and funding acquisition. All authors contributed to the article and approved the submitted version.

## References

[1] Hickel J. Outgrowing growth: why quality of life, not GDP, should be our measure of success [WWW Document]. The Correspondent (2020). Available online at: https://thecorrespondent.com/357/outgrowing-growth-why-quality-of-life-not-gdp-should-be-our-measure-of-success/413218170519-b4d036a5 (accessed March 25, 2021).

[2] Eurostat. Quality of life indicators - measuring quality of life - Statistics Explained [WWW Document]. Eurostat Stat. Explain (2021). Available online at: https://ec.europa.eu/eurostat/statistics-explained/SEPDF/cache/30610.pdf (accessed January 25, 2022).

[3] OECD. How's life? 2020: Measuring Well-Being. (2020). Available online at: https://www.oecd-ilibrary.org/content/publication/9870c393-en

[4] Šanda M Křupka J. Quality of life evaluation as decision support in public administration for innovation and regions development. Adm. SI Manag. Public. (2018) 2018:51–66. 10.24818/amp/2018.30-04

[5] Schalock R Baker A Claes C Gonzalez J Malatest R Loon J. The use of quality of life scores for monitoring and reporting, quality improvement, and research: quality of life outcomes. J Policy Pract Intellect Disabil. (2018) 15:176–82. 10.1111/jppi.12250

[6] Curmi S. The impact of urban development policies on quality-of-life in Malta : a case study on Marsascala (2017). Available online at: https://www.um.edu.mt/library/oar/handle/123456789/27305

[7] Rogge N Nijverseel I. Quality of life in the European Union: a multidimensional analysis. Soc Indic Res. (2019) 141:765–89. 10.1007/s11205-018-1854-y

[8] Somarriba N Espina P. Quality of life in the European Union: an econometric analysis from a gender perspective. Soc Indic Res. (2019) 142. 10.1007/s11205-018-1913-4

[9] Kim HS. Patterns of economic development: correlations affecting economic growth and quality of life in 222 countries. Polit Policy. (2017) 45:83–104. 10.1111/polp.12190

[10] Camargo Lemos D Ramírez P Fermino R. Individual and environmental correlates to quality of life in park users in Colombia. Int J Environ Res Public Health. (2017) 14. 10.3390/ijerph14101250 29048373 PMC5664751

[11] Koramaz E Türkoglu H. Measuring and understanding urban parks' contribution to quality of life in Istanbul. Soc Indic Res. (2018) 138:1–17. 10.1007/s11205-017-1657-6

[12] Omodior O Ramos W. Social determinants of health-related quality of life: a recreation setting analysis. Health Promot Pract. (2019) 21:152483991982757. 10.1177/1524839919827572 30786790

[13] Li C-L. Quality of life: the perspective of urban park recreation in three Asian cities. J Outdoor Recreat Tourism. (2020) 18:100260. 10.1016/j.jort.2019.100260

[14] Nieuwenhuijsen MJ. Green Infrastructure and Health. Annu Rev Public Health. (2021) 42:317–28. 10.1146/annurev-publhealth-090419-102511 33317317

[15] Holt E Lombard Q Best N Smiley Smith S Quinn J. Active and passive use of green space, health, and well-being amongst university students. Int J Environ Res Public Health. (2019) 16:424. 10.3390/ijerph16030424 30717193 PMC6388138

[16] Stigsdotter U Ekholm O Schipperijn J Toftager M Kamper-Jørgensen F Randrup T. Health promoting outdoor environments - associations between green space, and health, health-related quality of life and stress based on a Danish national representative survey. Scand J Public Health. (2010) 38:411–7. 10.1177/1403494810367468 20413584

[17] Chu Y-T Li D Chang P-J. Effects of urban park quality, environmental perception, and leisure activity on well-being among the older population. Int J Environ Res Public Health. (2021) 18:11402. 10.3390/ijerph182111402 34769914 PMC8583094

[18] de Jong K Albin M Skärbäck E Grahn P Björk J. Perceived green qualities were associated with neighborhood satisfaction, physical activity, and general health: results from a cross-sectional study in suburban and rural Scania, southern Sweden. Health Place. (2012) 18:1374–80. 10.1016/j.healthplace.2012.07.001 22889998

[19] Shafer CS Lee BK Turner S. A tale of three greenway trails: user perceptions related to quality of life. Landsc Urban Plan. (2000) 49:163–78. 10.1016/S0169-2046(00)00057-8

[20] Kothencz G Blaschke T. Urban parks: visitors' perceptions versus spatial indicators. Land Use Policy. (2017) 64:233–44. 10.1016/j.landusepol.2017.02.012

[21] Leslie E Sugiyama T Kremer P. Perceived and objectively measured greenness of neighbourhoods: are they measuring the same thing? Landsc. Urban Plan. (2010) 95:28–33. 10.1016/j.landurbplan.2009.11.002

[22] Chen C Luo W Li H Zhang D Kang N Yang X. Impact of perception of green space for health promotion on willingness to use parks and actual use among young urban residents. Int J Environ Res Public Health. (2020) 17:5560. 10.3390/ijerph17155560 32752166 PMC7432496

[23] Roe JJ Thompson CW Aspinall PA Brewer MJ Duff EI Miller D. Green space and stress: evidence from cortisol measures in deprived urban communities. Int J Environ Res Public Health. (2013) 10:4086–103. 10.3390/ijerph10094086 24002726 PMC3799530

[24] Shuda Q Bougoulias ME Kass R. Effect of nature exposure on perceived and physiologic stress: a systematic review. Complement Ther Med. (2020) 53:102514. 10.1016/j.ctim.2020.102514 33066853

[25] Tyrväinen L Ojala A Korpela K Lanki T Tsunetsugu Y Kagawa T. The influence of urban green environments on stress relief measures: a field experiment. J Environ Psychol. (2014) 38:1–9. 10.1016/j.jenvp.2013.12.005

[26] Van den Berg AE Jorgensen A Wilson ER. Evaluating restoration in urban green spaces: does setting type make a difference? Landsc. Urban Plan. (2014) 127:173–81. 10.1016/j.landurbplan.2014.04.012

[27] Kondo MC Fluehr JM McKeon T Branas CC. Urban green space and its impact on human health. Int J Environ Res Public Health. (2018) 15:445. 10.3390/ijerph15030445 29510520 PMC5876990

[28] Huang TTK Wyka KE Ferris EB Gardner J Evenson KR Tripathi D. The Physical Activity and Redesigned Community Spaces (PARCS) Study: protocol of a natural experiment to investigate the impact of citywide park redesign and renovation. BMC Public Health. (2016) 16:1160. 10.1186/s12889-016-3822-2 27842531 PMC5109670

[29] Bann CM Kobau R Lewis MA Zack MM Luncheon C Thompson WW. Development and psychometric evaluation of the public health surveillance well-being scale. Qual Life Res. (2012) 21:1031–43. 10.1007/s11136-011-0002-9 21947657

[30] Veitch J Salmon J Carver A Timperio A Crawford D Fletcher E. A natural experiment to examine the impact of park renewal on park-use and park-based physical activity in a disadvantaged neighbourhood: the REVAMP study methods. BMC Public Health. (2014) 14:600. 10.1186/1471-2458-14-600 24924919 PMC4073813

[31] Otero Peña JE Kodali H Ferris E Wyka K Low S Evenson KR. The role of the physical and social environment in observed and self-reported park use in low-income neighborhoods in New York City. Front Public Health. (2021) 9:656988. 10.3389/fpubh,0.2021.656988 33959584 PMC8095666

[32] Cohen S Kamarck T Mermelstein R. A global measure of perceived stress. J Health Soc Behav. (1983) 24:385–96. 10.2307/2136404 6668417

[33] Gunzler D Chen T Wu P Zhang H. Introduction to mediation analysis with structural equation modeling. Shanghai Arch Psychiatry. (2013) 25:390–4. 24991183 10.3969/j.issn.1002-0829.2013.06.009 PMC4054581

[34] Deng L Yang M Marcoulides KM. Structural equation modeling with many variables: a systematic review of issues and developments. Front Psychol. (2018) 9:580. 10.3389/fpsyg.2018.00580 29755388 PMC5932371

[35] VanderWeele TJ Vansteelandt S. Mediation analysis with multiple mediators. Epidemiol Methods. (2014) 2:95–115. 10.1515/em-2012-0010 25580377 PMC4287269

[36] Allison PD. Missing data techniques for structural equation modeling. J Abnorm Psychol. (2003) 112:545–57. 10.1037/0021-843X.112.4.545 14674868

[37] Enders C Bandalos D. The relative performance of full information maximum likelihood estimation for missing data in structural equation models. Struct Equ Model- Multidiscip J. (2001) 8:430–57. 10.1207/S15328007SEM0803_5

[38] R Core Team. R: A Language Environment for Statistical Computing. Vienna: R Foundation for Statistical Computing. (2019). Available online at: https://www.R-project.org/

[39] Arbuckle JL. Amos (Version 26.0) [Computer Program] (2019).

[40] Grilli G Mohan G Curtis J. Public park attributes, park visits, and associated health status. Landsc Urban Plan. (2020) 199:103814. 10.1016/j.landurbplan.2020.103814

[41] Umberson D Montez JK. Social relationships and health: a flashpoint for health policy. J Health Soc Behav. (2010) 51:S54–66. 10.1177/0022146510383501 20943583 PMC3150158

[42] Yim J. Therapeutic benefits of laughter in mental health: a theoretical review. Tohoku J Exp Med. (2016) 239:243–9. 10.1620/tjem.239.243 27439375

[43] Harber VJ Sutton JR. Endorphins and exercise. Sports Med. (1984) 1:154–71. 10.2165/00007256-198401020-00004 6091217

[44] Hegadoren KM O'Donnell T Lanius R Coupland NJ Lacaze-Masmonteil N. The role of β-endorphin in the pathophysiology of major depression. Neuropeptides. (2009) 43:341–53. 10.1016/j.npep.2009.06.004 19647870

[45] Kubryak OV Umriukhin AE Emeljanova IN Antipova OS Guseva AL Pertsov SS. Increased β-endorphin level in blood plasma as an indicator of positive response to depression treatment. Bull Exp Biol Med. (2012) 153:758–60. 10.1007/s10517-012-1819-0 23113278

[46] Bertrand E Smadja C Mauborgne A Roques BP Daugé V. Social interaction increases the extracellular levels of [Met]enkephalin in the nucleus accumbens of control but not of chronic mild stressed rats. Neuroscience. (1997) 80:17–20. 9252217 10.1016/s0306-4522(97)00136-x

[47] Baik J-H. Stress and the dopaminergic reward system. Exp Mol Med. (2020) 52:1879–90. 10.1038/s12276-020-00532-4 33257725 PMC8080624

[48] Mowen AJ Barrett A Pitas N Graefe AR Taff BD Godbey G. Americans' use and perceptions of local park and recreation services: results from an updated study. J Park Recreat Adm. (2018) 36:8861. 10.18666/JPRA-2018-V36-I4-8861

[49] Cerletti P Eze IC Keidel D Schaffner E Stolz D Gasche-Soccal PM. Perceived built environment, health-related quality of life and health care utilization. PLoS ONE. (2021) 16:e0251251. 10.1371/journal.pone.0251251 33956884 PMC8101743

[50] Hedblom M Gunnarsson B Iravani B Knez I Schaefer M Thorsson P. Reduction of physiological stress by urban green space in a multisensory virtual experiment. Sci Rep. (2019) 9:10113. 10.1038/s41598-019-46099-7 31300656 PMC6625985

[51] White MP Alcock I Wheeler BW Depledge MH. Would you be happier living in a greener urban area? A fixed-effects analysis of panel data. Psychol Sci. (2013) 24:920–8. 10.1177/0956797612464659 23613211

[52] Alcock I White MP Wheeler BW Fleming LE Depledge MH. Longitudinal effects on mental health of moving to greener and less green urban areas. Environ Sci Technol. (2014) 48:1247–55. 10.1021/es403688w 24320055

[53] Wang R Helbich M Yao Y Zhang J Liu P Yuan Y. Urban greenery and mental wellbeing in adults: cross-sectional mediation analyses on multiple pathways across different greenery measures. Environ Res. (2019) 176:108535. 10.1016/j.envres.2019.108535 31260914

[54] Giannico V Spano G Elia M D'Este M Sanesi G Lafortezza R. Green spaces, quality of life, and citizen perception in European cities. Environ Res. (2021) 196:110922. 10.1016/j.envres.2021.110922 33639147

[55] Larson L Jennings V Cloutier S. Public parks and wellbeing in urban areas of the United States. PLoS ONE. (2016) 11:e0153211. 10.1371/journal.pone.0153211 27054887 PMC4824524

[56] Fongar C Aamodt Randrup T Solfjeld I. Does perceived green space quality matter? Linking Norwegian adult perspectives on perceived quality to motivation and frequency of visits. Int J Environ Res Public Health. (2019) 16:2327. 10.3390/ijerph16132327 31266246 PMC6651101

[57] Costanza R Hart M Kubiszewski I Talberth J. A short history of GDP: moving towards better measures of human well-being. Solut J. (2016). Available online at: https://thesolutionsjournal.com/2016/02/22/a-short-history-of-gdp-moving-towards-better-measures-of-human-well-being/

[58] Costanza R Hart M Posner S Talberth J. Beyond GDP: The Need for New Measures of Progress. Boston, MA: Pardee Cent Study Longer-Range Future (2009). p. 39.

[59] Kapoor A Debroy B. GDP Is not a measure of human well-being. Harv Bus Rev. (2019). Available online at: https://hbr.org/2019/10/gdp-is-not-a-measure-of-human-well-being

[60] Rudolf R. Changing paradigms in measuring national well-being: how does Korea rank “beyond GDP”? Asian Soc Work Policy Rev. (2020) 14:118–121. 10.1111/aswp.12195

[61] Stiglitz JE. GDP is the wrong tool for measuring what matters. Sci Am. (2020) 323:24–31. Available online at: https://www.scientificamerican.com/article/gdp-is-the-wrong-tool-for-measuring-what-matters/10.1038/scientificamerican0820-24 39014767

[62] Adhikari B Pokharel S Mishra SR. Shrinking urban greenspace and the rise in non-communicable diseases in south Asia: an urgent need for an advocacy. Front Sustain Cities. (2019) 1:5. 10.3389/frsc.2019.00005

[63] Asri AK Lee H-Y Wu C-D Spengler JD. How does the presence of greenspace related to physical health issues in Indonesia? Urban For Urban Green. (2022) 74:127667. 10.1016/j.ufug.2022.127667

[64] Labib SM Shuvo FK Browning MHEM Rigolon A. Noncommunicable diseases, park prescriptions, and urban green space use patterns in a global south context: the case of Dhaka, Bangladesh. Int J Environ Res Public Health. (2020) 17:3900. 10.3390/ijerph17113900 32486391 PMC7313456

[65] Barboza EP Cirach M Khomenko S Iungman T Mueller N Barrera-Gómez J. Green space and mortality in European cities: a health impact assessment study. Lancet Planet Health. (2021) 5:e718–30. 10.1016/S2542-5196(21)00229-1 34627476

[66] Rojas-Rueda D Nieuwenhuijsen MJ Gascon M Perez-Leon D Mudu P. Green spaces and mortality: a systematic review and meta-analysis of cohort studies. Lancet Planet Health. (2019) 3:e469–77. 10.1016/S2542-5196(19)30215-3 31777338 PMC6873641

[67] Akaraci S Feng X Suesse T Jalaludin B Astell-Burt T. A systematic review and meta-analysis of associations between green and blue spaces and birth outcomes. Int J Environ Res Public Health. (2020) 17:2949. 10.3390/ijerph17082949 32344732 PMC7215926

[68] Barton J Rogerson M. The importance of greenspace for mental health. BJPsych Int. (2017) 14:79–81. 10.1192/S2056474000002051 29093955 PMC5663018

[69] White MP Elliott LR Grellier J Economou T Bell S Bratman GN. Associations between green/blue spaces and mental health across 18 countries. Sci Rep. (2021) 11:8903. 10.1038/s41598-021-87675-0 33903601 PMC8076244

[70] Jonker MF van Lenthe FJ Donkers B Mackenbach JP Burdorf A. The effect of urban green on small-area (healthy) life expectancy. J Epidemiol Community Health. (2014) 68:999–1002. 10.1136/jech-2014-203847 25053616

[71] Zhang Y Mavoa S Zhao J Raphael D Smith M. The association between green space and adolescents' mental well-being: a systematic review. Int J Environ Res Public Health. (2020) 17:6640. 10.3390/ijerph17186640 32932996 PMC7557737

[72] World Health Organization. Urban Green Spaces and Health. A Review of Evidence. Copenhagen: WHO Regional office for Europe (2016).

[73] Kruize H van der Vliet N Staatsen B Bell R Chiabai A Muiños G. Urban green space: creating a triple win for environmental sustainability, health, and health equity through behavior change. Int J Environ Res Public Health. (2019) 16:4403. 10.3390/ijerph16224403 31717956 PMC6888177

[74] Chen K Lin H You S Han Y. Review of the impact of urban parks and green spaces on residence prices in the environmental health context. Front Public Health. (2022) 10:993801. 10.3389/fpubh.2022.993801 36159304 PMC9490231

[75] Hunter RF Cleland C Cleary A Droomers M Wheeler BW Sinnett D. Environmental, health, wellbeing, social and equity effects of urban green space interventions: a meta-narrative evidence synthesis. Environ Int. (2019) 130:104923. 10.1016/j.envint.2019.104923 31228780

[76] Zenk SN Pugach O Ragonese-Barnes M Odoms-Young A Powell LM Slater SJ. Did playground renovations equitably benefit neighborhoods in Chicago? J Urban Health. (2021) 98:248–58. 10.1007/s11524-020-00472-4 32875485 PMC8079586

[77] New York City Department of Parks Recreation. Community Parks Initiative [WWW Document] (2015). Available online at: https://www.nycgovparks.org/about/framework-for-an-equitable-future/community-parks-initiative (accessed February 4, 2022).

